# *e*-Methanation with a spiral catalyst: optimized thermal management and long-term stability†

**DOI:** 10.1039/d4ra08236b

**Published:** 2025-03-07

**Authors:** Ryo Watanabe, Kohki Nishide, Hiroyasu Suganuma, Priyanka Verma, Hiroshi Akama, Choji Fukuhara

**Affiliations:** a Department of Applied Chemistry and Biochemical Engineering, Graduate School of Engineering, Shizuoka University 3-5-1 Johoku, Chuo-ku Hamamatsu Shizuoka 432-8561 Japan watanabe.ryo@shizuoka.ac.jp; b Department of Chemistry, Indian Institute of Technology Delhi Hauz Khas New Delhi – 110016 India

## Abstract

This study explores the development of a Joule-heated reaction field utilizing an electrically driven spiral-shaped catalyst for efficient CO_2_ methanation. Infrared thermal imaging in an uninsulated reactor reveals a rapid and uniform temperature rise along the spiral structure. With a 10 W input, CO_2_ conversion reached 80%, while 75% conversion was maintained even at 5 W. The catalyst's twist angle played a crucial role in optimizing heat transfer and CO_2_ conversion by enhancing swirl flow. Long-term stability tests at 5 W demonstrated sustained methane production over 50 hours at 350 °C, highlighting the catalyst's durability and energy efficiency.

## Abstract 2

The electrical-heated spiral catalyst shows stable performance for CO_2_ methanation for 50 hours, demonstrating the system's robustness.

## Graphical abstract

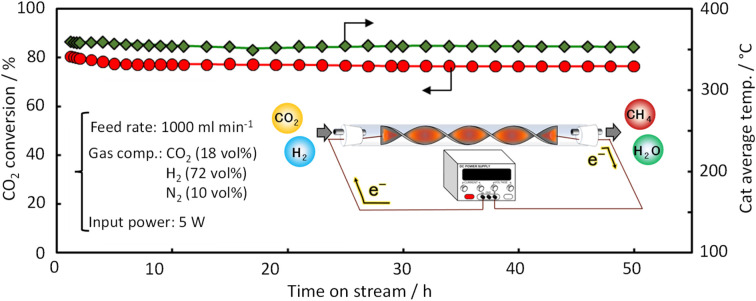

## 1. Introduction

The CO_2_ methanation reaction (CO_2_ + 4H_2_ → CH_4_ + 2H_2_O, Δ*H*^0^_298K_ = −165 kJ mol^−1^) has attracted considerable attention in recent years as a carbon capture utilisation (CCU) technology, facilitating the conversion of the greenhouse gas CO_2_ into valuable methane (CH_4_) resources.^1–3^ This technology enables CO_2_ reduction and efficient utilisation without reliance on fossil fuels by using hydrogen produced from renewable energy sources, thus contributing to global warming mitigation. Therefore, various catalytic systems for this reaction have been researched both domestically and internationally. CO_2_ hydrogenation is a multi-product reaction, potentially yielding not only CH_4_ but also methanol (CH_3_OH), higher hydrocarbons and alcohols, depending on the catalyst type and reaction conditions. Selective CH_4_ production *via* methanation is a thermodynamically favourable route at relatively low temperatures, whereas methanol synthesis (CO_2_ + 3H_2_ → CH_3_OH + H_2_O) requires precise catalyst design to suppress excessive hydrogenation to methane. Meanwhile, the Fischer–Tropsch synthesis pathway (CO_2_ + H_2_ → hydrocarbons + H_2_O) can produce long-chain hydrocarbons and oxygenates under certain catalytic and reaction conditions. Controlling the selectivity of CO_2_ hydrogenation products is therefore a critical factor in optimising the desired process for energy and chemical production. Tada *et al.* initiated the selective CO_2_ methanation earlier than others, demonstrating the effectiveness of Ni-based catalysts,^4,5^ while the superior performance of Ru-based catalysts has also been widely reported.^6–9^ The reaction mechanism and catalytic system developments have been extensively investigated.

Our research group is focused on the social implementation of the CO_2_ methanation system for industrial flue gases.^10,11^ Industrial exhaust gas usually contains oxygen, raising concerns about catalyst degradation from oxidation; however, we found that the presence of oxygen is effective for CO_2_ methanation. The coexistence of oxygen in the CO_2_ methanation reaction triggers an auto-methanation (AM) phenomenon that does not require external heating.^12–14^ In the AM process, combustion by hydrogen and oxygen causes internal heating in the reactor, which is the driving force for the methanation reaction, thereby achieving efficient CO_2_ conversion. This internal heating process is particularly effective in improving the energy efficiency of the reaction system and offers significant advantages over conventional external heating methods, with expected energy savings and simplification of the reactor. Furthermore, reusing the methane produced in this process as fuel could form an energy cycle, thus supporting a sustainable society. There is a major innovation in this technology. Spiral-shaped catalysts are commonly used in catalyst systems,^15^ as their configuration enables the thermal energy from hydrogen combustion to be efficiently supplied to the catalytic reaction field *via* swirl flow within the gas stream. The metallic helical base material facilitates heat transfer through the gas flow and conduction, further enhancing the reaction efficiency.

In this study, we explore a new internal heating method, an ‘electrical heating system’ (hereinafter referred to as *e*-reaction), where the catalyst itself is directly powered, generating reaction energy through Joule heat. Spiral-type catalysts typically use Ni–Cr metals, known for their relatively high resistance, allowing the formation of a heated reaction field upon electric current application. The shape of the catalyst system was also expected to play a significant role. As the methanation reaction is a highly exothermic reaction, heating the catalyst through energisation produces substantial reaction heat. This method achieves efficient and uniform temperature distribution within the reactor, potentially allowing for lower power inputs and rapid reaction field formation. Haldor Topsøe and the Technical University of Denmark are also developing similar systems using simpler reaction fields in the form of circular tubes.^16–18^ In recent years, systems utilizing Joule heating for catalytic reactions have been increasingly reported. For example, a reactor employing Rh/Al_2_O_3_-coated foam with Joule heating has achieved near-equilibrium conversion under high space velocity conditions.^19^ Additionally, a fully electrified eRWGS™ system using ohmic heating and a Ni catalyst at 1050 °C and 10 barg has been reported, successfully producing syngas (H_2_/CO = 2.0) without methane. Moreover, CH_4_-mediated RWGS has demonstrated higher activity and improved carbon suppression.^20^ Furthermore, Dong *et al.* have developed a programmable heating and quenching (PHQ) method using Joule heating, which enables high conversion while suppressing side reactions. This method has been successfully applied to methane pyrolysis and Ru-catalyzed ammonia synthesis, resulting in reduced coke formation, improved catalyst stability, and enhanced energy efficiency. Thus, PHQ offers significant potential for process intensification and decarbonization.^21^ Building upon these advancements, our proposed systems are designed to enhance heat and mass transfer by utilizing a spiral-shaped substrate, further improving catalytic performance and energy efficiency.

This research presents a breakthrough by overcoming the heat transfer and mass limitations of simple circular tube-type catalytic fields. By integrating them with an *e*-reaction system, this study enables a more efficient CO_2_ conversion process. In particular, when applying this system to CO_2_ methanation, we define it as ‘*e*-Methanation,’ distinguishing it from other CO_2_ conversion processes. This study reports the characterisation of the *e*-reaction system and the effect of twisting angle (which is a feature of spiral-shaped catalysts), and the viability of the system for extended reaction times, emphasizing its potential for social implementation.

## 2. Experimental

### 2.1. Preparation of structured catalyst

An impregnation and a washcoat method was used to prepare the spiral-shaped catalysts. The Ru component (loading amount: 10 wt%, source: Ru(NO_3_)_3_, Tanaka precious metals Co. Ltd) and CeO_2_ (Catalysis Society of Japan, labeled as JRC-CEO-2) were firstly added to distilled water and stirred well for 2 h at room temperature. Thereafter, the Ru component was loaded on the support by using the evaporation-to-dryness method at 80 °C by stirring. After drying, the powder obtained was calcined to 500 °C at the rate of 10 °C min^−1^ and kept at this temperature for 3 h before naturally being cooled to room temperature, thereby forming a Ru-loaded CeO_2_ catalyst. BET surface area was 119 m^2^ g_cat_^^−1^^, as shown in Fig. S1† (adsorption and desorption ithothem). The powder catalyst was then suspended in distilled water, and a spiral substrate (11 mm wide × 180 mm long) twisted to 720° was immersed in the suspension and dried overnight. The spiral base material, Celmet® (#7) manufactured by Toyama Sumitomo Electric, is a porous metal with continuous pores. This spiral base material was dipped and dried several times to obtain a spiral-shaped catalyst with a coating weight of 1.0 g·per piece. To study the effect of substrate twisting on CO_2_ methanation, catalysts were prepared with substrates twisted to 0° (plate-type catalyst), 360°, 720°, and 1080°.

### 2.2. Evaluation of catalytic performance

**Fig. 1:**
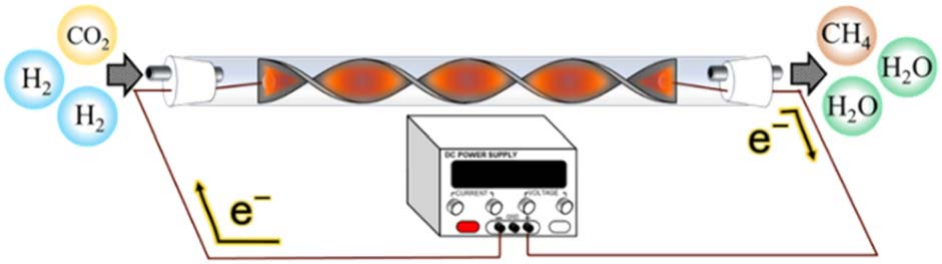
Schematic of the reaction system with a spiral-structured Ru/CeO_2_ catalyst.

A flow-type reactor was used to evaluate the catalytic properties. A spiral-shaped catalyst, connected to copper wires at both ends was installed in the reactor. Fig. 1 shows a schematic diagram of the reaction system using the spiral-type structured Ru/CeO_2_ catalyst. The copper wire and substrate were connected to the centre of both ends of the spiral shape using silver brazing. The catalyst underwent hydrogen reduction at 200 °C for 60 min, after which the temperature of the reaction field was lowered to room temperature. The temperature was subsequently increased by applying 10 W of power in an N_2_ atmosphere. When a constant temperature was reached, the reaction gas was supplied at CO_2_/H_2_/N_2_ = 10/40/50 vol% (total flow rate: 1.0 L min^−1^). The power was gradually decreased when the reaction reached a steady state. The influence of the reaction gas composition in this *e*-reaction system was also investigated at CO_2_/H_2_/N_2_ = 15/60/25 vol%, 18/72/10 vol% and 20/80/0 vol% (total flow rate: 1.0 L min^−1^). Furthermore, durability was conducted under reaction gas conditions of CO_2_/H_2_/N_2_ = 18/72/10 vol% (total flow rate: 1.0 L min^−1^).

## 3. Results and discussion

### 3.1. Temperature of reaction field

**Fig. 2:**
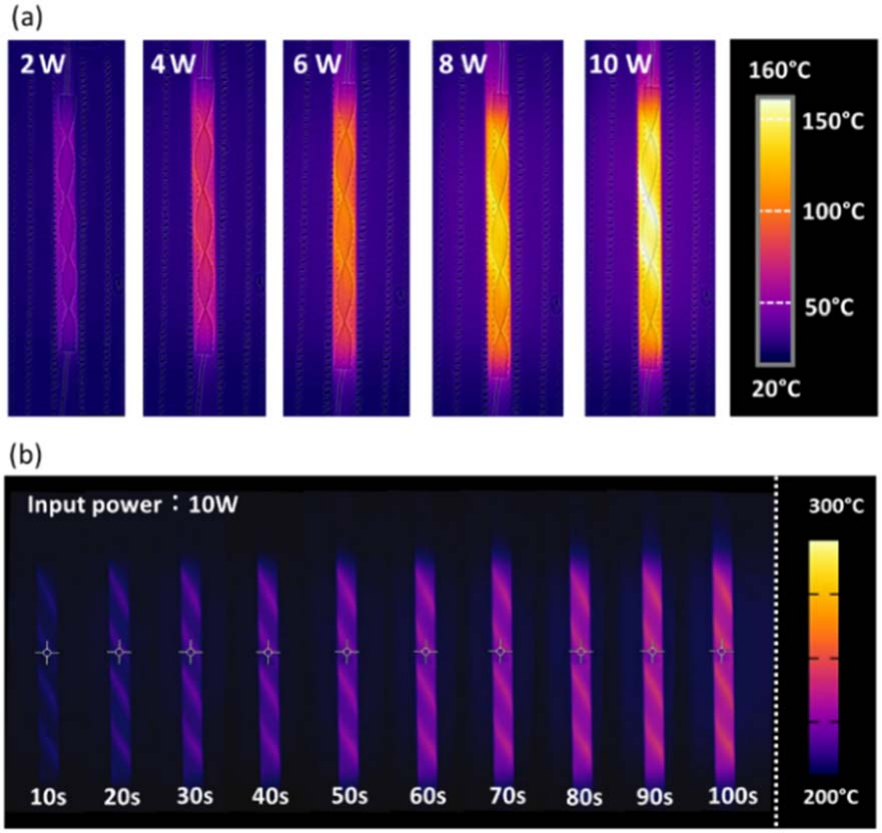
Infrared thermal images of the Ru(10)/CeO_2_-structured catalyst (720° twist): (a) under varying input power levels from 2 W to 10 W, and (b) at a constant input power of 10 W.

The feasibility of forming a heated reaction field by applying electric power to a spiral-shaped catalyst was investigated. In this study, the reactor was opened to obtain infrared thermal images, allowing heat to dissipate freely. Fig. 2(a) shows the IR image of a spiral-shaped catalyst with twist angle of 720°. The thermal image of this catalyst is shown for each power input condition in an N_2_ atmosphere. The resulting thermal profile reflected the spiral shape. Fig. 2(b) shows an IR thermal image of the catalyst heated at a fixed power of 10 W. The catalyst was rapidly heated with a 10 W input, reaching 220 °C after 60 s and 250 °C after 100 s, indicating a high thermal response. Fig. S2† shows the IR images of catalysts with different twist angles. While no significant differences are observed in the images, it appears that the catalyst with the twisted substrate shows a slightly higher gas-phase temperature increase.

**Fig. 3:**
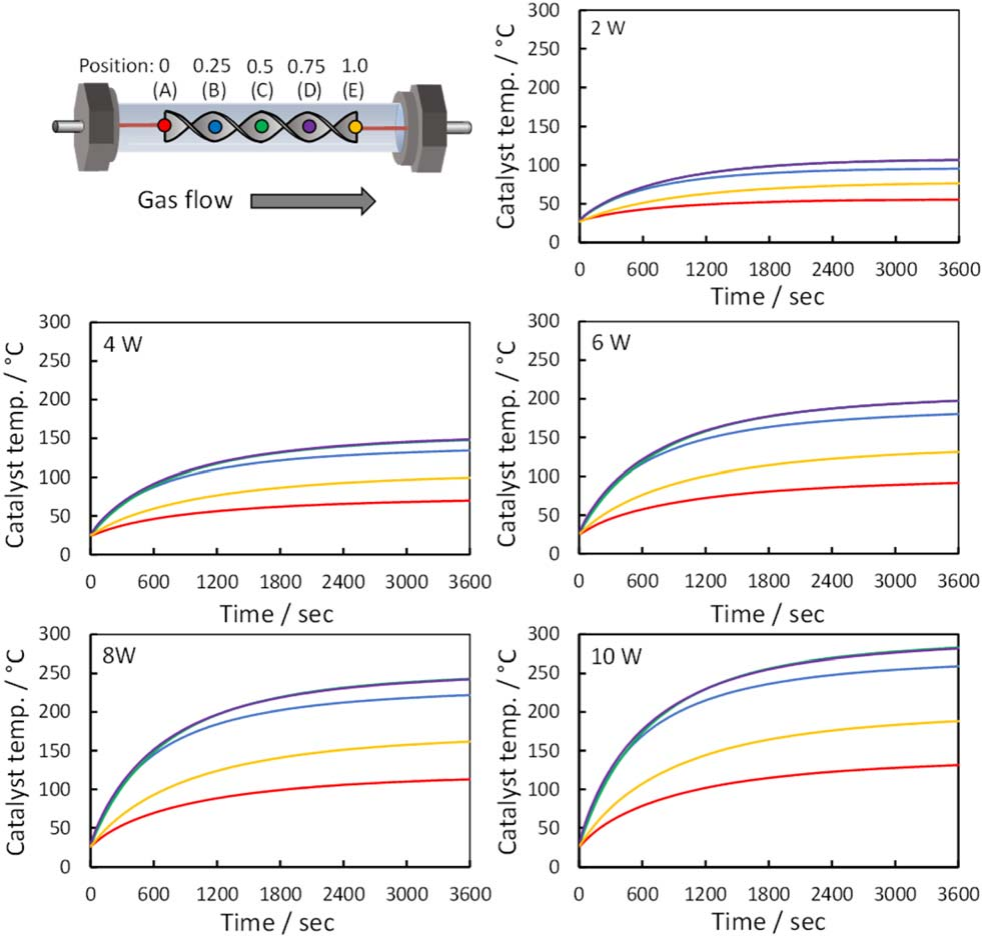
Temperature variation across five catalyst positions (0, 0.25, 0.50, 0.75, and 1.0), as the power output was varied from 2 W to 10 W. The positions of the lines correspond to the catalyst positions shown in the upper-left corner, with each line color matching its respective position.

To simulate an enclosed actual catalytic reaction field, temperature measurements were recorded within a closed reactor, minimizing heat loss from Joule heating. The temperatures of the reactor were measured at five points along the outside of the quartz tube where the spiral catalyst was installed. The dimensionless distance from the catalyst inlet to the outlet was defined as 1. Fig. 3 shows the temperature variations at five catalyst positions (0, 0.25, 0.50, 0.75, and 1.0) as power output varied. At catalyst position 0, the temperature was lower than that at other positions, between 0.25 and 1.0, due to the room-temperature N_2_ gas input. With increasing power, the temperature across the reaction field was increased, exceeding 200 °C under 10 W conditions at all positions except position 0 and 1.0, which would promote CO_2_ methanation.

### 3.2. CO_2_ methanation by electrically heated spiral catalyst

**Fig. 4:**
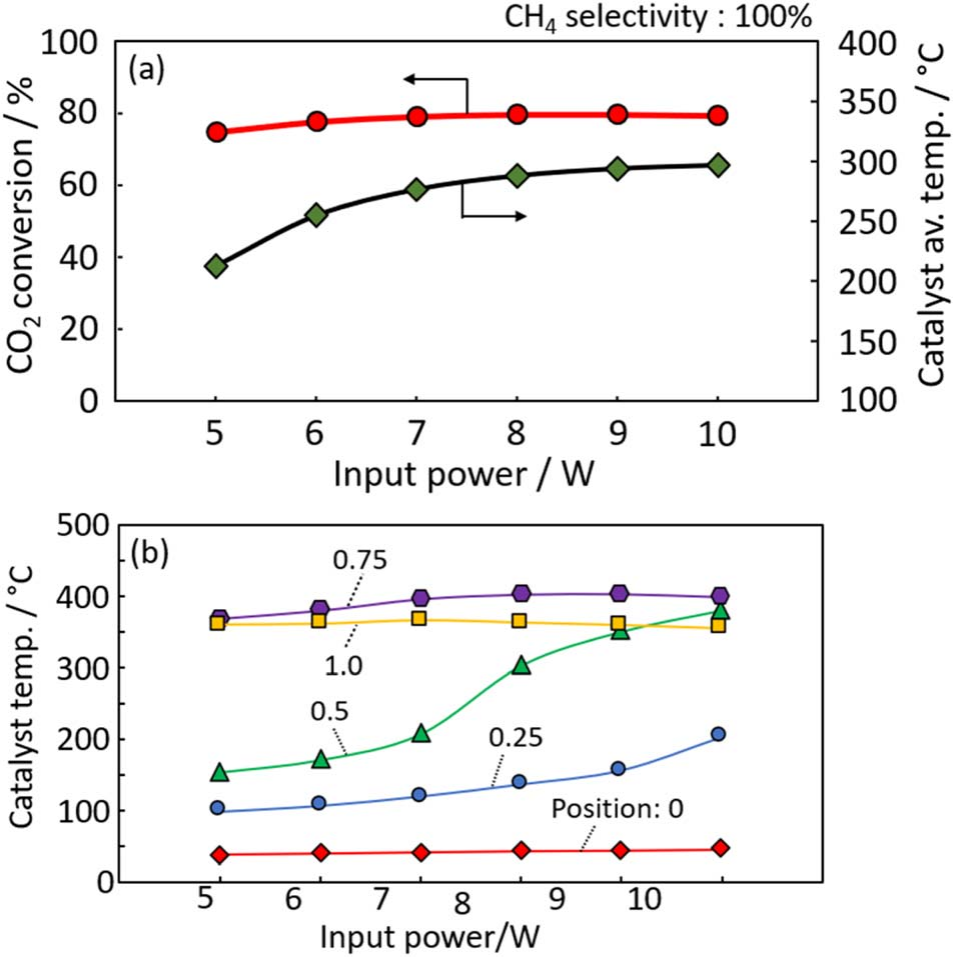
(a) CO_2_ conversion and (b) average temperature of spiral structured catalyst at an initial power input of 10 W.

Fig. 4(a) shows the CO_2_ conversion and average temperature of the spiral-structured catalyst when the power input, initially 10 W, was gradually reduced to 5 W. The average measured temperature at each position (expressed as the average temperature) is shown in the figure. At 10 W, the CO_2_ conversion reached 80% with an average temperature of 297 °C. Subsequently, the power input was gradually reduced. Even at 5 W, the CO_2_ conversion remained high at 75%, with an average temperature of 210 °C, approximately 90 °C lower than at 10 W. Throughout these conditions, the methane selectivity remained 100%, indicating that no byproducts such as CO or higher hydrocarbons were formed. These results show that high CO_2_ conversion power can be achieved with lower power consumption than required for CO_2_ electrolysis. Fig. 4(b) shows temperature measurements outside the quartz tube at each catalyst position over time as power output varied. At lower power, the temperature of the catalyst on the more outlet side is relatively high. This suggests that CO_2_ methanation occurs on the catalyst at the outlet side.

**Fig. 5:**
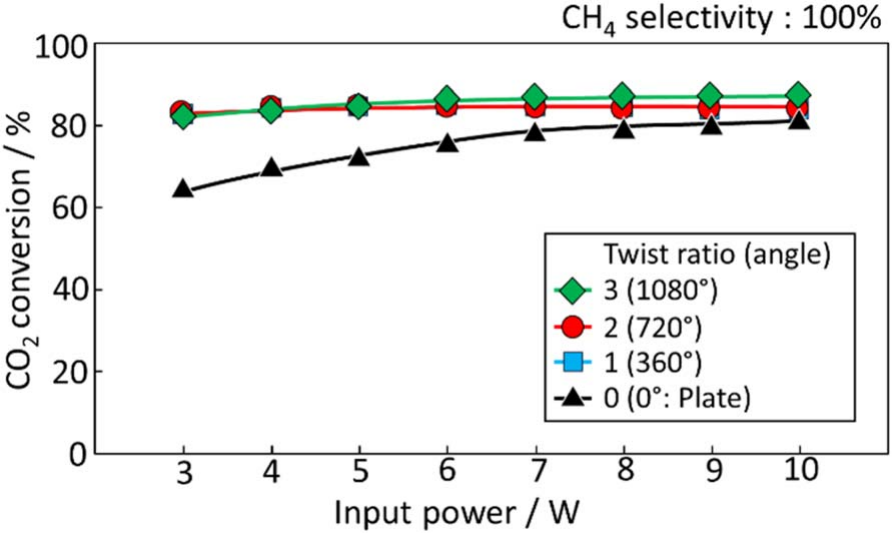
CO_2_ conversion of spiral structured catalyst at an initial power input of 10 W.

Fig. 5 shows the effect of varying the twist angle (0°–1080°) on methane conversion properties for plate and spiral catalysts. By increasing twist angles from 0° to 360°, CO_2_ conversion was increased, likely due to stronger swirl flow on the catalyst surface. Spiral heat exchangers are effective heat conductors, with their geometry promoting swirl flow, reducing boundary film resistance, and increasing the heat transfer coefficient. The induced turbulent gas flow thins the boundary layer, a major factor in increasing heat exchange efficiency. Therefore, higher twist angles increase methanation efficiency by lowering gas boundary film, which acts as thermal resistance. The fact of no significant difference in reactivity with increasing angle is predicted to indicate that the swirling effect is sufficient at 360° of twist angle. It is expected that the effect of twist angle would be demonstrated under conditions where the effect of mass transfer is significant.

**Fig. 6:**
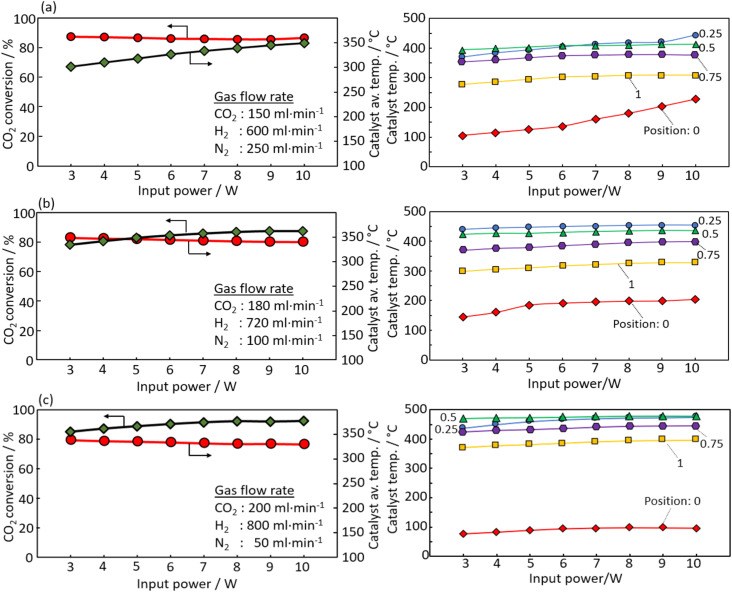
Effect of CO_2_ feed rate in the feedstock for *e*-Methanation under (a) CO_2_: 150 ml min^−1^, (b) CO_2_: 180 ml min^−1^, and (c) CO_2_: 200 ml min^−1^.

Fig. 6(a)–(c) show the effect of the CO_2_ concentration in the feedstock using a spiral catalyst with a twist ratio of 720°. The CO_2_ concentrations were varied at 150, 180 and 200 ml min^−1^, with a total flow rate of 1000 ml min^−1^. Higher CO_2_ concentrations maintained higher conversion rates even at lower power inputs. In particular, at a CO_2_ concentration of 200 ml min^−1^, conversion remained around 80% at a 3 W power input. This correlation is attributed to the increased heat generation from methanation as the CO_2_ supply increases, leading to higher temperatures at each catalyst position. Fig. 6(d)–(f) show the catalyst temperatures across power output at CO_2_ flow rates of 150, 180, and 200 ml min^−1^, showing minimal temperature reduction at the respective positions, likely due to efficient heating of the catalytic reaction field from heat generation under high gas flow conditions. In all conditions, temperatures were higher in the area behind position 0. This suggests that the reaction field is in the back part. As the CO_2_ supply increases, the temperature drop was small even under low power conditions. This is thought to be due to the fact that the temperature drop at the outlet of the catalyst, which is the reaction field, is suppressed because the heat generation rate was increased with the increase in the CO_2_ supply.

### 3.3. Durability performance of spiral structured catalyst for *e*-Methanation

**Fig. 7:**
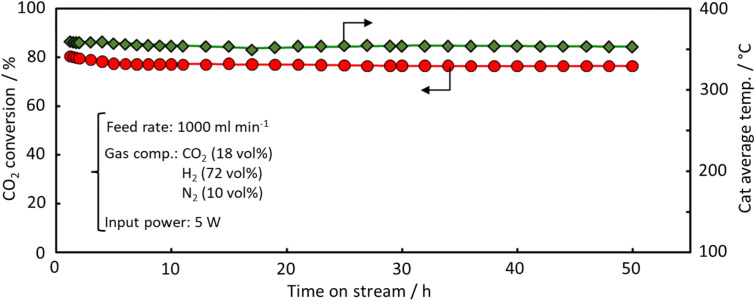
Durability performance of the spiral-structured catalyst for *e*-Methanation.

**Fig. 8:**
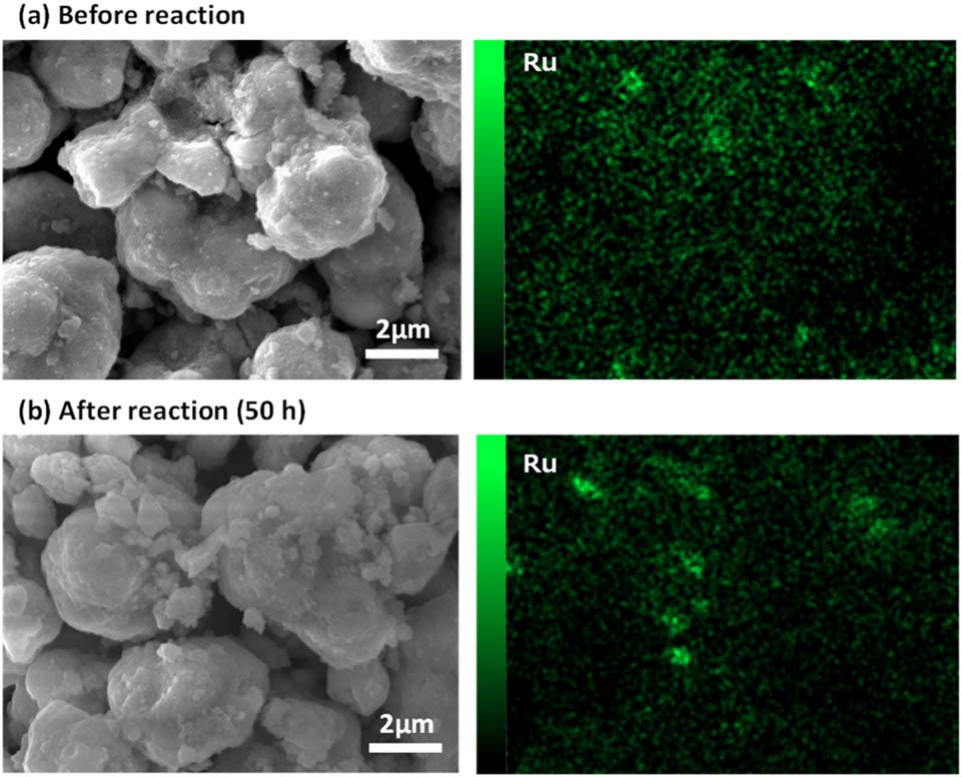
SEM images and Ru mapping of spiral-type Ru/CeO_2_ catalyst (a) before and (b) after reaction.

Fig. 7 shows the results of the *e*-Methanation over an extended period, with power input fixed at 5 W and the reaction characteristics evaluated after 50 h. In the early stages of the reaction, a slight decrease in conversion was observed, but the performance remained almost constant. The average temperature of the reaction field was maintained at 350 °C. This consistent temperature, maintained by the effective current flow through the substrate and the swirling flow generated by the spiral shape of the catalyst bed, provided sufficient downstream heating of the catalyst bed and supported high methane conversion characteristics under power-saving conditions. Fig. S3† shows the XRD data before and after the reaction. No significant differences were observed, although a slight crystal growth of CeO_2_ was noted after 50 hours of reaction. Similarly, Fig. 8 presents the SEM images and Ru mapping, with no major changes observed. There was no significant growth of the active component, Ru, suggesting that the catalyst demonstrates high durability. The results indicate that the developed spiral-shaped electrically powered catalyst maintains stable CO_2_ methanation performance over extended operation, demonstrating both high energy efficiency and durability. The consistent temperature distribution enabled by Joule heating and enhanced heat transfer *via* swirl flow effectively sustains methane conversion, even under reduced power input conditions. A key innovation of this study lies in overcoming the limitations of conventional electrically heated catalytic systems, such as simple tube reactors. By optimizing the catalyst's spiral configuration, this system achieves superior heat and mass transfer, ensuring effective thermal management without external heating elements. The observed stability in both structural integrity and catalytic activity further confirms the viability of this approach for long-term operation. These findings underscore the potential for practical implementation of *e*-Methanation technology. The ability to maintain efficient CO_2_ conversion with minimal energy input positions this system as a promising solution for industrial carbon recycling. Moreover, the inherent scalability of the electrically powered spiral catalyst system offers new possibilities for integrating renewable energy sources into methanation processes, contributing to the advancement of carbon–neutral energy systems.

## 4. Conclusions

This study demonstrates the feasibility of an electrically heated spiral catalyst for CO_2_ methanation. The spiral catalyst exhibited a rapid thermal response and efficient heating across various power inputs, reaching temperatures above 200 °C at 10 W, sufficient to drive methanation reactions. The CO_2_ conversion rate reached 80% at 10 W and maintained high conversions (75%) even at a reduced input of 5 W, indicating the energy efficiency of the system compared with traditional CO_2_ electrolysis. The twist angle of the spiral catalyst played a critical role in enhancing methanation performance, with the highest CO_2_ conversion observed at a 1080° twist due to improved gas swirling and reduced thermal resistance from boundary films. Additionally, the system demonstrated high tolerance to increased CO_2_ concentrations, maintaining high conversion even at low power inputs. Long-term durability tests at 5 W over 50 h showed stable performance with a consistent reaction field temperature, confirming the robustness and efficiency of the catalyst in prolonged operations. These findings highlight the potential of the spiral-structured catalyst as an energy-efficient and scalable approach for CO_2_ methanation, contributing to sustainable carbon utilization strategies. The system's adaptability to varying CO_2_ concentrations and power inputs suggests its applicability in decentralized power-to-gas systems and integration with renewable energy sources. Future research should focus on optimizing catalyst materials to enhance reaction kinetics further, investigating system performance under dynamic power fluctuations, and exploring scale-up strategies for industrial implementation.

## Data availability

The data that support the findings of this study are available from the corresponding author, Ryo Watanabe, upon reasonable request.

## Conflicts of interest

There are no conflicts to declare.

## Supplementary Material

RA-015-D4RA08236B-s001
