# Supplementary material for: e-Methanation with a spiral catalyst: optimized thermal management and long-term stability
Source: RSC Adv. 2025 Mar 7;15(10):7365–70. doi: 10.1039/d4ra08236b (PMC11886956; doi:10.1039/d4ra08236b)

Supplementary Information (SI) for RSC Advances.
This journal is © The Royal Society of Chemistry 2025

Supporting information

Title: *e*-Methanation with a spiral catalyst: optimized thermal management and long-term stability

Authors: Ryo Watanabe[a,*], Kohki Nishide[a], Hiroyasu Suganuma[a], Priyanka Verma[b], Hiroshi Akama[a], Choji Fukuhara[a]

[a] *Department of Applied Chemistry and Biochemical Engineering, Graduate School of Engineering, Shizuoka University, 3-5-1 Johoku, Chuo-ku, Hamamatsu, Shizuoka 432-8561*

[b] *Department of Chemistry, Indian Institute of Technology Delhi, Hauz Khas, New Delhi – 110016, India*

Figure S-1 Adsorption-Desorption Isotherms of as-prepared spiral-type Ru/CeO_2 catalyst.

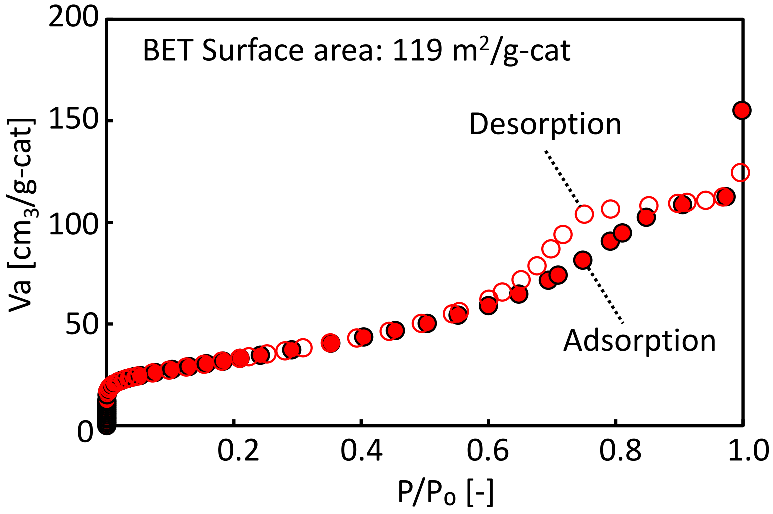

Figure S-2 Effect of twist angle differences in spiral-shaped catalysts on reaction field temperature distribution evaluated by IR thermal imaging.

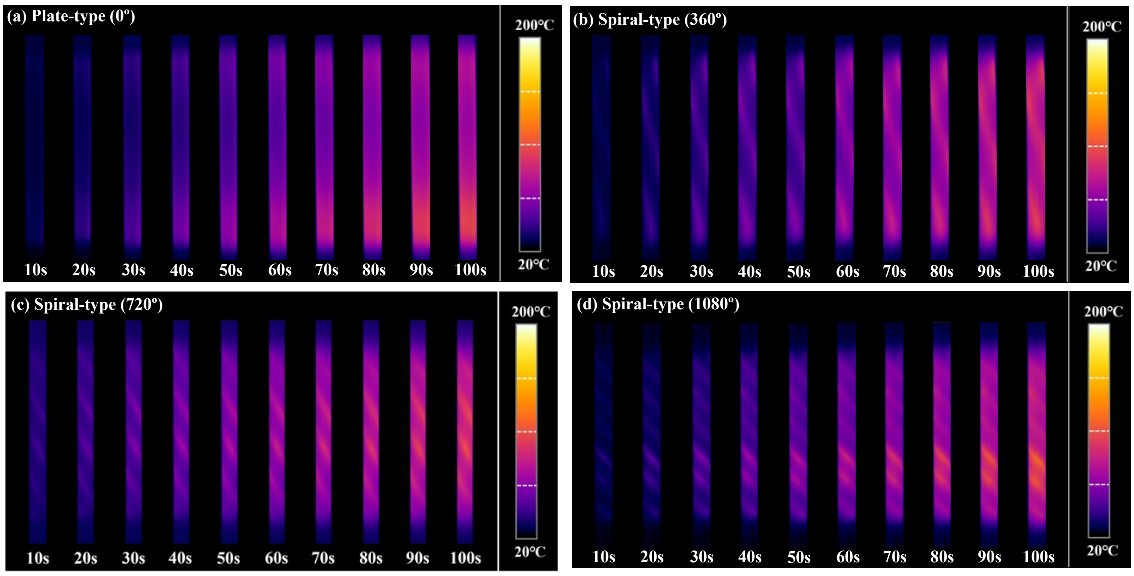

Figure S-3 XRD patterns of spiral-type structured Ru/CeO_2 catalyst before and after reaction.

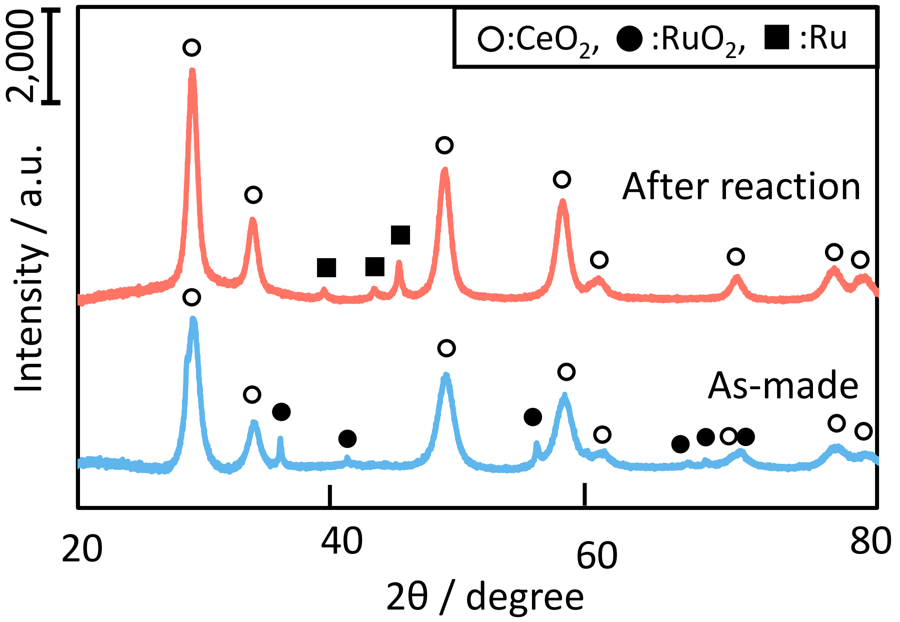

Supplement: RA-015-D4RA08236B-s001 [file RA-015-D4RA08236B-s001.pdf]
